# Intravenous Cyclophosphamide Therapy for Anti-IFN-Gamma Autoantibody-Associated *Mycobacterium abscessus* Infection

**DOI:** 10.1155/2018/6473629

**Published:** 2018-12-30

**Authors:** Ploenchan Chetchotisakd, Siriluck Anunnatsiri, Ratanavadee Nanagara, Arnone Nithichanon, Ganjana Lertmemongkolchai

**Affiliations:** ^1^Division of Infectious Diseases and Tropical Medicine, Department of Medicine, Faculty of Medicine, Khon Kaen University, Khon Kaen, Thailand; ^2^Division of Rheumatology, Department of Medicine, Faculty of Medicine, Khon Kaen University, Khon Kaen, Thailand; ^3^Cellular and Molecular Immunology Unit, Centre for Research and Development of Medical Diagnostic Laboratories (CMDL), Faculty of Associated Medical Sciences, Khon Kaen University, Khon Kaen, Thailand

## Abstract

**Introduction:**

Anti-interferon-gamma (IFN-*γ*) autoantibodies are increasingly recognized as a cause of adult-onset immunodeficiency (AOID) worldwide. These patients are susceptible to various intracellular pathogens especially nontuberculous mycobacteria. Most of the patients have a refractory clinical course. Herein, we report the use of immunotherapy with pulse intravenous cyclophosphamide (IVCY) in patients who had progressive, refractory *Mycobacterium abscessus* infection.

**Method:**

We included patients, seen at Srinagarind Hospital, Thailand, infected with *M. abscessus*, who had received ≥3 courses of parenteral antibiotics within the last 12 months and who received pulse IVCY with a tapering dose of prednisolone.

**Results:**

There were 8 AOID patients who met the criteria and received pulse IVCY between January 2011 and December 2015. One patient was lost to follow-up after 5 courses of IVCY: he had died at home 3 months later. Five patients had favorable outcomes: 2 were able to discontinue NTM therapy, and 3 had stable disease and were on NTM treatment without hospitalization for parenteral antibiotics. Two patients relapsed and needed hospitalization. The IFN-*γ* Ab titers among the 7 patients were significantly decreased during treatment, and the median initial antibody titer started at 200,000 and then decreased to 5,000 after 2 years of treatment (*P* < 0.0001). The antibody titer reduction among responsive vs. nonresponsive patient was significantly different after 6 months of treatment: the median antibody titer was 5,000 and 100,000, respectively (*P* = 0.0467).

**Conclusion:**

IVCY therapy might be an alternative treatment for AOID patients infected with *M. abscessus* and refractory to antimycobacterial therapy.

## 1. Introduction

Anti-interferon-*γ* (IFN-*γ*) autoantibodies are increasingly recognized as a cause of adult-onset immunodeficiency (AOID) worldwide [1–19]. These patients are susceptible to various intracellular pathogens especially nontuberculous mycobacteria (NTM). The majority of patients have a persistent NTM infection and require long-term antimycobacterial therapy [20]. Some patients infected with rapidly growing mycobacteria (RGM) have recurrent disease despite continuous oral antimycobacterial treatment and need hospitalization for parenteral imipenem. Various adjunctive therapies have been used to treat patients with anti-IFN-*γ* autoantibodies [3, 4, 21–25], including B cell depletion therapy with rituximab which has attracted much attention. Owing to economic constraints, rituximab is not accessible through the Thai National Health Insurance program for off-label treatment.

Cyclophosphamide is an alkylating agent affecting depletion of both B and T cells, hence reducing the production of pathogenic autoantibodies. Intravenous cyclophosphamide (IVCY) has been used successfully as an induction therapy for severe lupus nephritis, either 6 monthly pulses of IVCY (NIH regimen) [26] or 6 pulses of lower-dose IVCY every 2 weeks (Euro-Lupus Nephritis Trail) [27]. The low-dose regimen was found to be associated with half as many severe infections as compared to the high-dose regimen, and long-term outcomes did not differ at the 10-year follow-up [28]. Currently, IVCY has been used as a standard regimen particularly for severe autoimmune and autoinflammatory diseases such as systemic lupus erythematosus (SLE) [29–32], active alveolitis from systemic sclerosis [33–35], antineutrophil cytoplasmic antibodies- (ANCA-) associated primary vasculitis syndrome [36–38], and other autoimmune diseases [39–44].

Plasmapheresis followed by pulse cyclophosphamide has been successfully treated in an AOID patient with recurrent MAC infection [21]. Herein, we report the use of immunotherapy with intravenous cyclophosphamide (IVCY) in 8 AOID patients with a high titer of anti-IFN-*γ* autoantibodies who had progressive refractory *M. abscessus* infection needing frequent hospitalization for parenteral antibiotics.

## 2. Methods

### 2.1. Ethics

This study is a retrospective study and has been approved by our ethics committee (HE601199).

### 2.2. Patients and Immunotherapy

All patients were seen at Srinagarind Hospital, a tertiary care university hospital. The standard regimen for treating AOID patients infected with RGM is a combination of oral antimycobacterial agents, mostly macrolides and fluoroquinolones. If they have progressive disease—despite being continuously on oral antimycobacterial treatment—they will be hospitalized for parenteral antibiotic treatment (i.e., particularly imipenem for 2–4 weeks).

We included patients who received ≥3 courses of parenteral antibiotics within 12 months and who received pulse IVCY. The regimen consists of methylprednisolone 1,000 mg intravenous on the last day of parenteral antibiotics followed by oral prednisolone 30 mg/day and IVCY 400 mg every 2 weeks for 6 cycles, then IVCY 400 mg every 4–6 weeks for 3 cycles plus oral prednisolone 15 mg/day, then IVCY 400 mg every 8–12 weeks for 3 cycles and oral prednisolone 10 mg/day, and then IVCY 400 mg every 12 weeks and oral prednisolone 5 mg/day until 2 years of therapy was completed. All patients received a combination of oral antimycobacterial treatment continuously.

### 2.3. Clinical Monitoring

Patients underwent routine safety monitoring during the IVCY therapy, including complete blood count with differentials, urine analysis, and renal and hepatic function chemistries. We also monitored their anti-IFN-*γ* titer. Disease activity was assessed by observing clinical signs and evidence of active infection on computed tomography, pathology, culture, or smear as indicated. Treatment and clinical data were collected by review of chart records.

### 2.4. Determination of IFN-*γ* Autoantibody Titers

A method for determining anti-IFN-*γ* autoantibody titer was previously described [20, 45]. Briefly, a 96-well polystyrene plate (Nunc) was precoated with 100 *μ*l of anti-human IFN-*γ* capture antibody (BD Biosciences) overnight at 4°C. On the day of the experiment, the precoated plate was washed 3 times with 0.05% Tween20 in phosphate-buffered saline (PBS) and then blocked with 200 *μ*l of 10% fetal bovine serum in PBS for 2 h at room temperature. Meanwhile, plasma samples were diluted with 300 pg/ml of recombinant human IFN-*γ* at 1 : 100, 1 : 1,000, 1 : 5,000, 1 : 10,000, 1 : 50,000, 1 : 100,000, 1 : 200,000, 1 : 400,000, 1 : 800,000, and 1 : 1,600,000 before being incubated at 37°C for 1 h. The preincubated diluted plasma samples (100 *μ*l) were added to the plate and incubated at room temperature for 1 h. After washing 5 times with 0.05% Tween20-PBS, a 100 *μ*l mixture of horseradish peroxidase (HRP), tagged streptavidin, and biotinylated anti-human IFN-*γ* detection antibody (BD Biosciences) was added to the plate and incubated at room temperature for 1 h. The plate was washed 7 times before adding 100 *μ*l of tetramethylbenzidine (TMB) substrate (BD Biosciences), which was incubated at room temperature for 15 min. The reaction was stopped by adding 25 *μ*l of 2 N H_2_SO_4_. Absorbance was measured at 450/570 nm by spectrophotometry.

The level of detectable human IFN-*γ* was calculated according to the standard curve from each plate. The percentage of neutralizing IFN-*γ* from each diluted plasma sample was calculated according to the equation below. The highest plasma dilution that resulted in ≥50% neutralization of the IFN-*γ* was considered a positive titer.

(1) $$\begin{array}{c} \%neutralizing\;IFN‐\gamma =\left(\frac{1-detectable\;IFN‐\gamma }{300}\right)\times 100 \end{array}$$

GraphPad Prism version 6 (GraphPad) was used for the statistical analysis. Statistical significance was determined using ANOVA with Dunnett's multiple comparisons test.

All the data used to support the findings of this study are included within the article.

## 3. Results

### 3.1. Patients

There were 8 AOID patients who met the criteria and received pulse IVCY between January 2011 and December 2015. All of the patients had *M. abscessus* lymphadenitis along with other organ involvements. All but two patients were coinfected with other opportunistic infections. All of the patients had had progressive mycobacterial infection for over 10–48 months. The median NTM infection was 17 months before IVCY therapy. The patients received 3-6 courses of parenteral antimicrobial (mostly imipenem) within 12 months prior to the IVCY.

Table 1 summarizes the clinical data and outcomes of disseminated *M. abscessus*-infected patients treated with IVCY. Patients received 5–25 cycles of IVCY (median, 17 cycles). There were no serious laboratory test results related to cyclophosphamide during the IVCY therapy. Patient #1 received 5 courses of IVCY and then was lost to follow-up: he had died at home some 3 months after we lost contact with him. (Patient #1 was censored from the analysis.) Among the 7 evaluable cases, 5 had favorable outcomes: 2 (patients #2 and #3) had complete remission and were able to discontinue the NTM therapy while 3 (patients #4, #5, and #6) had stable disease but remained on NTM treatment without the need of hospitalization for parenteral antibiotics. Two (patients #7 and #8) relapsed and needed hospitalization. Patient #7 had enlarged lymph nodes after 12 cycles of IVCY and needed hospitalization for imipenem; after which, he resumed IVCY for another 3 cycles but developed *Herpes zoster ophthalmicus* which required admission for administration of parenteral acyclovir. He had a persistent enlarged epitrochlear lymph node after 25 cycles of IVCY. Patient #8 had 2 relapses requiring hospitalization for parenteral imipenem.

### 3.2. Anti-IFN-*γ* Autoantibody Titers

**Figure 1:**
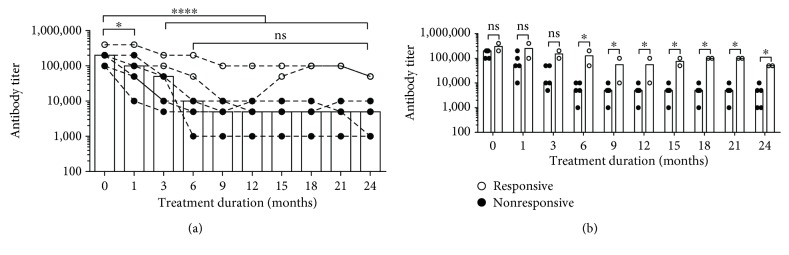
Anti-IFN-*γ* autoantibody titer changes in plasma from responsive patients (*n* = 5, close circle (●)) and nonresponsive patients (*n* = 2, open circle (○)) at different time points after receiving intravenous cyclophosphamide (IVCY) pulse therapy. (a) Align dot plot with bar graph represents the median anti-IFN-*γ* autoantibody titer changing dynamic at each time point in all 7 cases. (b) Scatter dot plot with bar graph compares the median titer between responsive and nonresponsive patients. Statistical significance was determined using ANOVA with Dunnett's multiple comparisons test; ns: nonsignificant; ^∗^*P* < 0.05 and ^∗∗∗∗^*P* < 0.0001.

The anti-IFN-*γ* Ab titers among the 7 evaluable patients were significantly decreased during treatment. The median initial antibody titer starting at 200,000 then was decreased to 5,000 after 2 years of treatment (*P* < 0.0001). The median antibody titers started to be significantly reduced 1 month after receiving IVCY (Figure 1). Antibody titer reduction among responsive patients (*n* = 5) vs. nonresponsive patients (*n* = 2) was significantly different after 6 months of treatment (median antibody titer was 5,000 and 100,000, respectively) (*P* = 0.0467). Antibody titer among the responsive patients started at between 100,000 and 200,000 and was reduced to around 5,000 (range, 1,000–10,000). The antibody titer of nonresponsive patients started at between 200,000 and 400,000 and was reduced to between 10,000 and 200,000 after the first year of treatment and to 100,000 after the second year of treatment.

**Figure 2:**
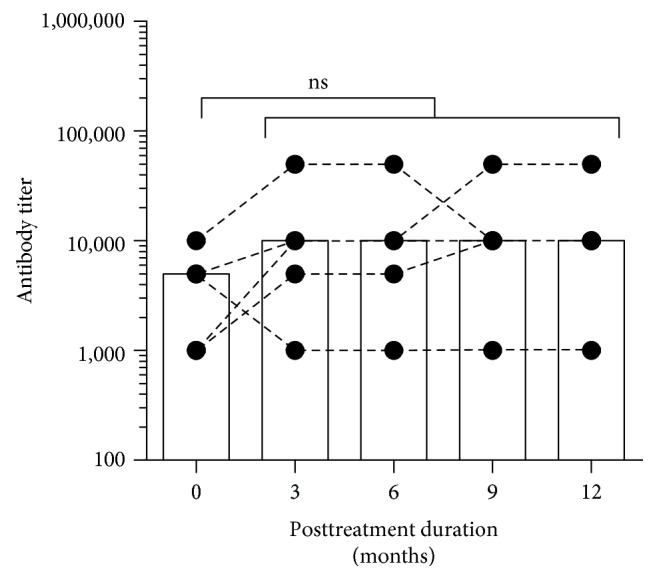
Anti-IFN-*γ* autoantibody titers of responsive patients after stopping IVCY pulse therapy. Median anti-IFN-*γ* autoantibody titers in plasma from responsive patients (*N* = 5) were followed for 1 year at different time points after stopping IVCY pulse therapy. Statistical significance was determined using ANOVA with Dunnett's multiple comparisons test; ns: nonsignificant.

Among the 5 responsive patients, their anti-IFN-*γ* autoantibody titers were followed for 1 year after completing the IVCY pulse therapy (Figure 2). The median anti-IFN-*γ* autoantibody titer had increased from 5,000 to 10,000 three months after discontinuation of treatment, albeit the difference was not statistically significant. The median titers remained stable for up to 12 months.

## 4. Discussion

Our patients had extensive disease as a result of *M. abscessus* infection, which progressed over the years: the median duration of infection was 17 months. All of the patients had lymphadenitis along with other sites of *M. abscessus* infection (Table 1). All but two had coinfection with other opportunistic infections. The patients were refractory to antimycobacterial treatment, so we sought an adjunctive immunotherapy. Rituximab has been used in AOID patients associated with mycobacterial infection with clinical improvement [22–25]; among 7 patients reported, they received 4-18 cycles of rituximab over 1-5 years of treatment. Since rituximab is not accessible in our setting, we tried to treat our AOID patients with an available immunosuppressant drug. We used an IVCY regimen resembling the Euro-Lupus protocol for lupus nephritis in combination with a single pulse of methyl prednisolone followed by a moderate dose of prednisolone for the first 3 months and maintenance with low-dose corticosteroid until the end of study. We treated only patients who had a protracted clinical course, defined as having received ≥3 courses of parenteral antibiotic within 12 months. We started the IVCY treatment regimen at the end of the parenteral therapy after the disease was controlled.

Among the 7 evaluable cases, 5 had a clinical response. To date, in two of our patients, the *M. abscessus* infection was cleared, and the patients were able to discontinue NTM treatment. Three of the remaining patients had stable disease and continued treatment for *M. abscessus* without needing to be hospitalized for parenteral therapy. Only 2 patients did not respond; they relapsed and needed hospitalization for imipenem therapy.

We demonstrated that IVCY reduced IFN-*γ* Ab titers significantly. Among the 7 patients studied, the median initial titers decreased after 2 years of treatment (*P* < 0.0001) and the titers started to decline within one month of receiving IVCY (Figure 1(a)). Comparing between responders and nonresponders, the reduction in Ab titers among responders was significantly greater than that among nonresponders after 6 months of therapy throughout the end of 2 years of therapy (Figure 1(b)). The IFN-*γ* titers among responders at the end of treatment ranged between 1,000 and 5,000 while for nonresponders' titers, they averaged 50,000. Our findings suggest that IFN-*γ* Ab titers are correlated to clinical response; thus, an early drop in IFN-*γ* titer is a predictor of a good outcome of IVCY treatment. The use of Ab titers to monitor disease activity or to determine when to discontinue antimycobacterial treatment needs further investigation.

Patients #7 and #8 received an extended course of IVCY (25 and 20 cycles, respectively). The lack of clinical improvement suggests that extension of IVCY treatment does not help nonresponders to reduce Ab titers to the level of a responsive titer. Among IVCY nonresponders, switching to other immunosuppressive drugs (i.e., mycophenolate mofetil, azathioprine, or calcineurin inhibitor) may be considered. Importantly, Ab titers among responders were stable up to one year after stopping IVCY therapy.

The present study had some limitations. First, it was a retrospective study, so some information might have been lost. Second, the study had a small sample size but that is partly because AOID is uncommon, and not all cases of anti-IFN-*γ* autoantibody-mediated disseminated NTM require immunotherapy. Third, we did not identify the isolates of *M. abscessus* to subspecies level nor identify an inducible macrolide resistance gene which might have effect on the treatment outcomes. Fourth, we did not perform plasma-mediated inhibition of IFN-*γ*-stimulated STAT-1 phosphorylation; notwithstanding, we previously demonstrated that plasma specimens containing anti-interferon-*γ* autoantibodies inhibited interferon-*γ*-induced STAT1 phosphorylation [1]. The Ab titers were correlated to clinical response; thus, we hypothesize that the IFN-*γ* antibodies in our patients were functioning. Despite these limitations, our results offer valuable clinical information vis-à-vis patients with anti-IFN-*γ* autoantibody-associated *M. abscessus* infection who had refractory disease in a resource-limited setting.

In conclusion, IVCY therapy might be an alternative adjunctive immunotherapy for AOID patients infected with *M. abscessus* and refractory to antimycobacterial treatment since it improved clinical outcomes and reduced the titer of anti-IFN-*γ* autoantibodies. A rapid decline in the titer of anti-IFN-*γ* antibody after 6 months of immunosuppressive treatment predicted a good outcome. In cases of a persistently high anti-IFN-*γ* antibody titer, rather than extending the use of IVCY, the clinician should consider switching to other immunosuppressive drug(s) with different target(s) of action. To avoid serious infection related to the use of long-term cyclophosphamide, a better outcome may be achieved by using a less toxic immunosuppressive drug.

## Figures and Tables

**Table 1:** Clinical data and outcomes among disseminated *M. abscessus*-infected patients treated with intravenous cyclophosphamide (IVCY).

Patient no.	Age/sex	Organ involvement	Treatment duration before IVCY (month)	Other opportunistic infection	Oral antibiotic	Parenteral antibiotic prior to IVCY/duration (week)	No. of IVCY cycle received	Initial IFN-*γ* Ab titer	IFN-*γ* Ab titer at the end of treatment	Follow-up after IVCY (month)	Outcome
1	52/M	Lymph nodes, lung, spleen	19	Tuberculosis lung, Herpes zoster, penicilliosis	Clarithromycin, ofloxacin	(1) Cefoxitin/2 (2) Imipenem/2 (3) Imipenem/4 (4) Imipenem/4	5	1 : 100,000	1 : 50,000	Not applicable	Unevaluable: lost to follow-up and died 3 months later

2	54/M	Lymph nodes, lung	15	None	Clarithromycin, ciprofloxacin	(1) Imipenem/2 (2) Imipenem/4 (3) Imipenem/4	14	1 : 200,000	1 : 5,000	57	Response: cured with discontinued NTM treatment

3	57/M	Lymph nodes, lung, liver	10	Tuberculosis lung	Azithromycin, ciprofloxacin	(1) Imipenem/4 (2) Imipenem/4 (3) Imipenem/4	16	1 : 200,000	1 : 1,000	44	Response: cured with discontinued NTM treatment

4	41/M	Lymph nodes, nasal septum	19	Melioidosis	Clarithromycin, ofloxacin	(1) Imipenem/2 (2) Imipenem/4 (3) Imipenem/4 (4) Imipenem/4	17	1 : 200,000	1 : 1,000	60	Response: stable on NTM treatment without hospitalization for parenteral antibiotic

5	54/F	Lymph nodes, skin	12	None	Clarithromycin, ciprofloxacin	(1) Imipenem/4 (2) Imipenem/4 (3) Imipenem/4 (4) Imipenem/4	17	1 : 100,000	1 : 1,000	28	Response: stable on NTM treatment without hospitalization for parenteral antibiotic

6	65/M	Lymph nodes, lung, femur	48	Salmonellosis	Clarithromycin, ciprofloxacin	(1) Imipenem/4 (2) Imipenem/4 (3) Imipenem/4	17	1 : 100,000	1 : 5,000	44	Response: stable on NTM treatment without hospitalization for parenteral antibiotic

7	34/M	Lymph nodes, lung, multiple bones	15	Cryptococcosis, tuberculosis bone	Clarithromycin, ciprofloxacin, linezolid	(1) Imipenem/2 (2) Imipenem/2 (3) Imipenem/4 (4) Imipenem/4 (5) Imipenem/4 (6) Imipenem/4	25	1 : 200,000	1 : 50,000	45	Not response: relapsed with hospitalization for parenteral antibiotic

8	20/F	Lymph nodes, skin, lung, liver, spleen, pancreas, humerus	26	Cryptococcosis, Herpes zoster	Azithromycin, ofloxacin, doxycycline, linezolid	(1) Imipenem/4 (2) Imipenem/4 (3) Imipenem/4	20	1 : 400,000	1 : 50,000	41	Not response: relapsed twice with hospitalization for parenteral antibiotic

## Data Availability

The data used to support the findings of this study are included within the article.
